# Short-Term Effects of Ketamine and Isoflurane on Left Ventricular Ejection Fraction in an Experimental Swine Model

**DOI:** 10.5402/2011/582658

**Published:** 2011-06-27

**Authors:** Benjamin Wessler, Christopher Madias, Natesa Pandian, Mark S. Link

**Affiliations:** New England Cardiac Arrhythmia Center, Tufts Medical Center, Boston, MA 02111, USA

## Abstract

*Background*. General anesthesia is an essential element of experimental medical procedures. Ketamine and isoflurane are agents commonly used to induce and maintain anesthesia in animals. The cardiovascular effects of these anesthetic agents are diverse, and the response of global myocardial function is unknown. *Methods*. In a series of 15 swine, echocardiography measurements of left ventricular ejection fraction (LVEF) were obtained before the animals received anesthesia (baseline), after an intramuscular injection of ketamine (postketamine) and after inhaled isoflurane (postisoflurane). *Results*. The mean LVEF of an unanesthetized swine was 47 ± 3%. There was a significant decrease in the mean LVEF after administration of ketamine to 41 + 6.5% (*P* = 0.003). The addition of inhaled isoflurane did not result in further decrease in mean LVEF (mean LVEF 38 ± 7.2%, *P* = 0.22). Eight of the swine had an increase in their LVEF with sympathetic stimulation. *Conclusions*. In our experimental model the administration of ketamine was associated with decreased LV function. The decrease may be largely secondary to a blunting of sympathetic tone. The addition of isoflurane to ketamine did not significantly change LV function. A significant number of animals had returned to preanesthesia LV function with sympathetic stimulation.

## 1. Introduction

Anesthesia is frequently required during the medical management of animals for both therapeutic procedures and experimental models. The response of the cardiovascular system to anesthetic agents can be highly variable, based on agent selection, dosing, and the experimental model [1–3]. Commonly used induction and maintenance anesthesia protocols involve agents that are known to effect the sympathetic and parasympathetic nervous systems, vascular tone, and contractile properties of the myocardium [4–6]. Ketamine, a derivative of phencyclidine and cyclohexamine, is a NMDA antagonist that is frequently used to induce anesthesia in swine experimental models. It acts on the thalamocortical, reticular activating, and limbic systems [7]. After induction, anesthesia maintenance is often achieved with the inhaled agent isoflurane, an halogenated ether that is one of the least cardiotoxic agents available [3]. Limited models exist to describe the complex cardiovascular effects of ketamine which include increased heart rate, cardiac output, and vascular resistance. A model using isolated chick embryo hearts suggests that this agent decreases myocardial contractile amplitude [8]. So too, there is limited information on the cardiovascular effects of isoflurane which has been suggested to decrease aortic pressures, cardiac output, stroke volume, and contractility [3, 9, 10]. Descriptions of the effects of these agents on global myocardial function are limited to isolated muscle preparations or different animal models [11, 12]. The specific effects of these agents on global systolic myocardial function in vivo have not been fully evaluated and are critical to understanding the physiologic responses to general anesthesia. The hypothesis of this work is that global myocardial systolic function is affected by both ketamine and isoflurane. In this analysis we use standard echocardiography techniques to evaluate the short-term changes in left ventricular ejection fraction after exposure to ketamine and isoflurane in a swine model. 

## 2. Methods

Young male domesticated swine, four to eight weeks old and weighing 8 to 12 kg, were used in this study. The research protocol was approved by the Animal Research Committee of Tufts Medical Center and conducted in accordance with the regulations of the Association for Assessment and Accreditation of Laboratory Animal Care.

The anesthesia protocol has been previously published [13]. Swine were sedated with intramuscular ketamine at a concentration of 12 milligrams per kilogram of body weight. Animals were then intubated and anesthetized with inhaled isoflurane which was maintained at 1.0 to 2.0 percent mixed with oxygen and nitrous oxide. Each animal was placed prone in a sling to approximate physiologic cardiac anatomy and hemodynamics. The anesthesia protocol and experimental design described here are part of a larger research program evaluating sudden death due to low-energy chest-wall impact (commotio cordis) [13].

Echocardiography was performed at the start of the study prior to ketamine administration, while the animal was being held on the stretcher (baseline). Views included parasternal short and long axis, and apical 2 and 4 chamber. Echocardiography was repeated 5 minutes after ketamine administration, and then again 5 minutes after isoflurane administration. After induction of ventricular fibrillation by ball impact and defibrillation, echocardiocardiography was repeated [13–15]. Heart rates were obtained at each echocardiographic study for each subject.

## 3. Data Analysis

Echocardiograms were reviewed by a single board certified echocardiographer (N.P.) who was blinded to swine specific data and to the stage of the experimental protocol. The global left ventricular ejection fraction (LVEF) was quantitatively assessed as well as the presence or absence of wall motion abnormalities. 

Results are presented as mean ± standard deviation unless otherwise specified. Data were analyzed using Two-Sample Independent *t *tests. Statistical significance was set at a probability value of *P* < 0.05 with a two sided confidence interval and assuming equal variance. The statistical analyses were run using SPSS Version 17.0. SPSS Inc., IBM Company, 233 S. Wacker Drive, Chicago, Illinois 60606.

## 4. Results

Fifteen swine with a mean weight of 9.9 ± 1.4 kg were used for this analysis. Prior to anesthesia the mean heart rate of the swine was 144 ± 17 bpm and the mean LVEF was 47 ± 3.2%. No wall motion abnormalities were identified at baseline (Tables 1 and 2). 

**Figure 1:**
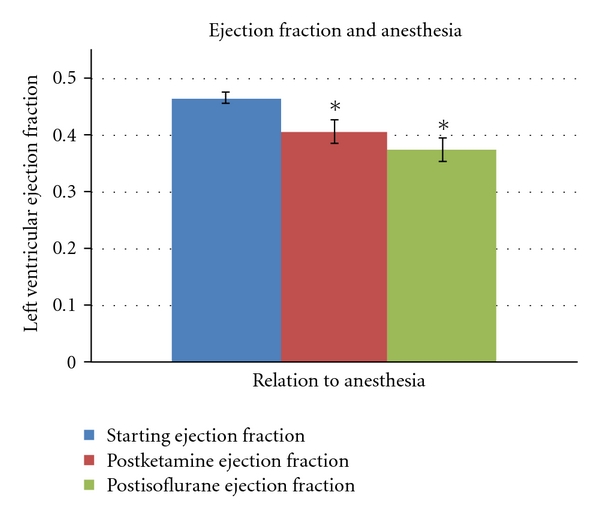
Mean left ventricular ejection fraction and relationship to anesthetic. Baseline (blue), postketamine sedation (red), and postisoflurane (green) left ventricular ejection fraction data are shown. Results are presented as mean LVEF ± standard deviation. Significant change from baseline EF is identified by *(*P* < 0.05).

**Figure 2:**
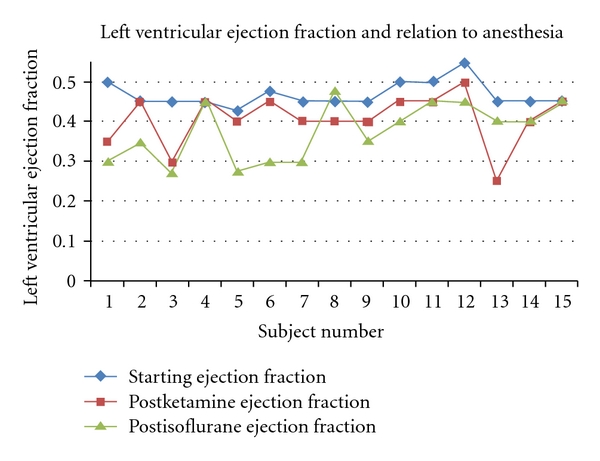
Left ventricular ejection fraction by individual subject at baseline (blue diamonds), postketamine (red squares), and postisoflurane (green triangles).

After ketamine the mean heart rate was unchanged at 147 ± 14 bpm (*P* = ns). As compared to baseline, there was a significant decrease in the mean LVEF to 41 ± 6.5%, (*P* = 0.003). A decrease in LVEF was observed in 12 of the 15 (80%) subjects, with 3 subjects showing no change in global left ventricular function (Figures 1 and 2). In these animals, the LVEF decreased by a mean value of 12.8 ± 13.0% from the baseline. There were no wall motion abnormalities evident after ketamine administration.

After administration of isoflurane, the mean heart rate dropped significantly to 132 ± 22 bpm (*P* = 0.03). Ten of the 15 (67%) subjects demonstrated an overall further decrease in LVEF with the addition of isoflurane, with the LVEF decreasing by a mean value of 19.6 ± 15.1% from the baseline. There was a slight overall further decrease in mean LVEF, but this value was not significantly lower than after ketamine administration (38 ± 7.2%, *P* = 0.22) (Tables 1 and 2). There was a single wall motion abnormality identified in the postisoflurane study for subject no. 2. This case demonstrated severe hypokinesis of the distal apical septum (Figures 1 and 2).

After initiation of ventricular fibrillation by baseball strikes to the chest wall and defibrillation, 7 of the 15 animals had a return to preanesthesia LVEF (Table 1).

## 5. Discussion

Ketamine and isoflurane are frequently used in experimental animal models to induce and maintain anesthesia. This is the first study to evaluate the short-term effects of these agents on global myocardial function in a live, whole animal model. 

Ketamine has significant and varied effects on cardiovascular physiology. The contractility and global LVEF changes documented in this swine model are consistent with a report by Schulte-Sasse et al. that showed short-term elevation of filling pressures with ketamine use in human subjects undergoing coronary artery bypass operations [16]. The data presented here suggest that this rise in filling pressure is largely related to a decrease in global left ventricular systolic function. Numerous reports in various animal models have worked to define these changes. In one dog model, for example, there appeared to be an overall increase in stress-related hormones [17]. Experiments on rats and guinea pigs have noted that ketamine tends to not only increase blood pressure and heart rate but also decrease left ventricular diastolic pressure. The latter changes have been linked to cation movement, specifically magnesium, and associated kinase pathways [18]. 

 Recent molecular work might offer further insight for the cardiodepressive effects of ketamine. Specifically, Kawano et al. have demonstrated in an in vitro model that ketamine induces inhibition of sarcolemmal K + ATP sensitive potassium channels [19]. Blockade of these channels has long been associated with decreased contractility [20]. Additionally, recent descriptions in a cat model demonstrate increased histamine release, which has also been linked to global cardiovascular depression [21]. 

There are limited data on the cardiovascular effects of isoflurane in animal models and even fewer reports of the cardiac response in humans. Early reports on human subjects suggest that there are minimal short-term myocardial depressive effects with isoflurane and that the pulmonary capillary wedge pressure does not significantly change [22, 23]. In contrast to these human reports, animal models have shown more substantial myocardial depressive effects. An echocardiographic study of rats anesthetized with various anesthetic agents showed that isoflurane resulted in a statistically significant decrease in left ventricular ejection fraction compared with conscious controls [24]. There is a recent swine model description of isoflurane-induced changes in myocardial function; however, echocardiographic parameters are not used. Instead, decreases in systolic function were measured with more invasive ultrasound techniques [25]. The results of our work are consistent with an overall minimal short-term myocardial depressive effect seen in humans with a trend towards the depressive effects seen in previous animal work. 

The molecular basis of isoflurane induced myocardial depression has long been postulated to relate to the regulation of calcium and its relationship to important contractile elements. Isoflurane decreases the amount of calcium present in the sarcoplasmic reticulum [26]. Additionally, there is evidence that this anesthetic directly inhibits the sarcolemmal calcium channels themselves [27]. These changes result in decreased concentrations of calcium being released and available to elements of contraction and might contribute to a decrease in global systolic function.

The overall short-term myocardial depressive effects of ketamine and the trend towards depressive effects of isoflurane on the swine myocardium have important experimental implications. These short-term changes should be considered when interpreting experimental hemodynamic changes. Additionally, while there have not been systematic evaluations of these agents and their short-term effects on human systolic function, it is reasonable to hypothesize that the human heart undergoes similar short-term changes when exposed to these agents. Lastly, arrhythmic, ischemic, and heart failure disease states in human research studies are all independently associated with decreasing left ventricular ejection fraction [28–30]. It is reasonable to interpret results from experimental models such as those within the framework of a somewhat reduced ejection fraction, not in the context of the starting, preanesthetic ejection fraction.

There are several limitations to this work. It should be noted that the baseline values in this study were obtained while the animals were excited as they were without anesthesia. As a result, these values may represent a baseline sympathetic-induced increase in ejection fraction [31]. Although it is possible that the change in ejection fraction after ketamine injection is due in part to a decrease from a baseline sympathetically elevated ejection fraction, the lack of significant change in heart rate suggests that the swine were unlikely to be significantly sympathetically activated. Importantly, the results here represent only short-term changes associated with these agents. The effects seen here do not allow comment on the long-term effects of ketamine and isoflurane on global systolic function. An additional limitation relates to the concentration of isoflurane used. In a dog experimental model there were significant decreases in contractility indices seen at isoflurane concentrations over 2%. These effects were not seen at concentrations of 1.25% [3]. The concentrations used in our experimental model were maintained in the 1% to 2% range. This might account for the observations of variable response, inconsistent change in ejection fraction, and a lack of statistically significant change with the addition of isoflurane. Specifically, the concentrations used here might have been occasionally below an unknown threshold value that is needed to induce depression of myocardial function.

## 6. Conclusion

 Ketamine use was associated with a significant short-term decrease in LV function in this experimental swine model. The decrease may be largely due to a blunting of sympathetic tone. The addition of isoflurane to ketamine did not significantly change global LV function. A significant number of animals had returned to preanesthesia LV function with subsequent sympathetic stimulation.

## Figures and Tables

**Table 1:** Animal Data. Ejection fraction (EF) measurements were made using standard echocardiography techniques. Pulse rate was recorded using standard ECG techniques and is presented as beats per minute. Pre-ket represents the starting values, Post-ket represents the values after ketamine was administered. Post-iso represents the values after isoflurane was administered. Postdefibrillation represents the values after induction of ventricular fibrillation and successful defibrillation. *Animals did not recover from ventricular fibrillation.

Animal no.	Pre-ket EF	Pre-ket pulse	Post-ket EF	Post-ket pulse	Post-iso EF	Post-iso pulse	Postdefibrillation EF	Postdefibrillation pulse
1	0.50	144	0.35	162	0.30	108	0.35	106
2	0.45	156	0.45	136	0.35	97	0.28	96
3	0.45	152	0.30	148	0.28	156	0.25	130
4	0.45	156	0.45	152	0.45	161	0.45	138
5	0.43	144	0.40	152	0.28	138	0.15	120
6	0.48	164	0.45	160	0.30	111	0.30	125
7	0.45	168	0.40	132	0.30	122	0.30	95
8	0.45	152	0.40	153	0.48	153	0.48	
9	0.45	100	0.40	120	0.35	132	0.45	119
10	0.50	128	0.45	144	0.40	136	0.48	131
11	0.50	132	0.45	152	0.45	121	*	74
12	0.55	132	0.50	136	0.45	112	0.55	138
13	0.45	136	0.25	176	0.40	163	*	82
14	0.45	156	0.40	144	0.40	157	0.50	71
15	0.45	136	0.45	136	0.45	112	0.55	61

**Table 2:** Hemodynamics. Values are mean ± SEM. LVEF is the left ventricular ejection fraction from blinding echocardiography. *Indicates *P* < 0.05 compared with baseline.

Parameters	Baseline	Postketamine	Postisoflurane
*N*	15	15	15
Heart rate, bpm	143.7 ± 17.2	146.9 ± 13.8	131.9 ± 22.0
LVEF	0.47 ± 0.01	0.41 ± 0.02*	0.38 ± 0.02*
